# The VIRTUE Index: A Novel Echocardiographic Marker Integrating Right–Left Ventricular Hemodynamics in Acute Heart Failure

**DOI:** 10.3390/jcm14248803

**Published:** 2025-12-12

**Authors:** Dan-Cristian Popescu, Mara Ciobanu, Diana Țînț, Alexandru-Cristian Nechita

**Affiliations:** 1 Department of Cardiology, Clinical Emergency Hospital “Sfântul Pantelimon”, 021652 Bucharest, Romania; popescu_dan_95@yahoo.com (D.-C.P.); mara2228@gmail.com (M.C.); cardionechita@yahoo.com (A.-C.N.); 2 Faculty of Medicine, “Carol Davila” University of Medicine and Pharmacy, 020021 Bucharest, Romania; 3 Faculty of Medicine, “Transilvania” University, 500036 Brașov, Romania; 4 Department of Cardiology, ICCO Clinics, 500059 Brasov, Romania

**Keywords:** acute heart failure, echocardiography, prognosis, Virtue Index, NT-proBNP, ventricular coupling

## Abstract

**Background/Objectives:** Acute heart failure (AHF) is a heterogeneous syndrome with phenotype-dependent prognosis. NT-proBNP is the reference biomarker, but standard echocardiographic measures (TAPSE, RV–RA gradient, LVOT VTI) offer only partial prognostic insight. The Virtue Index, defined as (RV–RA gradient)/(TAPSE × LVOT VTI), was introduced to integrate right–left ventricular interaction. This study evaluated its clinical and prognostic performance in AHF and its behavior across ejection-fraction phenotypes. **Methods:** We retrospectively analyzed 222 patients with AHF; complete data for Virtue calculation were available in 168 (99 HFrEF, 69 HFpEF) patients. HFmrEF patients were excluded from subgroup prognostic analyses. Correlation with NT-proBNP was assessed using Spearman testing with bootstrap intervals, and in-hospital mortality prediction was evaluated using ROC analysis with DeLong comparisons. **Results:** In HFpEF, the Virtue Index correlated moderately with NT-proBNP (ρ = 0.38, *p* = 0.002) and showed fair prognostic discrimination (AUC 0.704), similar to the RV–RA gradient (0.724) and higher than TAPSE or LVOT VTI. In HFrEF, correlation was weak (ρ = 0.19, *p* = 0.06) and predictive accuracy was modest (AUC 0.584), while LVOT VTI performed best (AUC 0.700). NT-proBNP outperformed all echocardiographic parameters in both groups. **Conclusions:** The Virtue Index reflects integrated hemodynamics and shows phenotype-dependent prognostic value in AHF, being more informative in HFpEF than in HFrEF. Although NT-proBNP remained superior, Virtue may complement biomarker-based risk assessment by offering a rapid, bedside estimate of short-term mortality risk.

## 1. Introduction

Acute heart failure (AHF) remains one of the most common cardiovascular emergencies, responsible for millions of hospital admissions each year and associated with high short-term mortality and rehospitalization rates [1,2]. The underlying mechanisms leading to this unfavorable outcome are complex, involving abrupt shifts in preload and afterload, neurohormonal activation, and variable contributions from both ventricles [3,4]. Importantly, prognosis is not uniform across phenotypes: patients with HFrEF differ in clinical course and outcomes from those with HFpEF [5,6].

Early risk stratification is essential to guide management decisions but remains challenging in routine practice. Clinical assessment alone has limited prognostic accuracy [7], and although biomarkers such as NT-proBNP are powerful diagnostic and prognostic tools [8,9,10], their availability may be delayed or restricted in some clinical contexts. Echocardiography provides immediate, bedside information and is central to the evaluation of both left and right ventricular function [11,12]. In routine clinical practice, echocardiographic evaluation in acute settings primarily focuses on left ventricular function, while right ventricular assessment may be more challenging and, at times, overlooked due to technical or time constraints. However, right ventricular performance plays a crucial role in determining outcomes in AHF. This rationale supports the development of a simple, rapid, and feasible index capable of simultaneously evaluating both ventricles at the bedside.

Several echocardiographic parameters have been linked to outcomes in AHF. The left ventricular outflow tract velocity–time integral (LVOT VTI), reflecting forward stroke volume, has shown prognostic relevance in both chronic and acute settings [13,14]. On the right side, tricuspid annular plane systolic excursion (TAPSE) and the tricuspid regurgitation–derived pressure gradient are established indices of right ventricular systolic performance and pulmonary pressures [15,16,17,18]. However, each parameter reflects only a limited dimension of cardiac function. Emerging evidence highlights the importance of ventricular interdependence, whereby right- and left-sided hemodynamics are dynamically coupled, influencing both symptoms and outcomes [19].

To capture this interaction, we previously introduced the Virtue Index as a composite marker defined as the ratio of the right ventricular to right atrial pressure gradient (RV–RA gradient) to the product of TAPSE and LVOT VTI [20]. In the present study, we evaluate its prognostic performance in a new cohort of acute heart failure patients and explore whether its utility differs between HFrEF and HFpEF. Although initial findings suggested that the index may offer incremental prognostic information by integrating RV load, longitudinal systolic function, and LV forward flow, validation remains limited, particularly regarding phenotype-specific discrimination based on ejection fraction.

These three components reflect complementary aspects of acute pump failure: TAPSE as a marker of RV longitudinal systolic function, the RV–RA pressure gradient as a surrogate of RV afterload, and LVOT VTI as an indicator of forward stroke volume. By integrating biventricular performance under acute loading conditions, the VIRTUE Index aims to capture hemodynamic compromise that may not be apparent through LVEF alone.

## 2. Materials and Methods

### 2.1. Study Design and Patient Population

We conducted a retrospective analysis including patients admitted with AHF between January 2024 and June 2025. The diagnosis of AHF was established in accordance with the 2021 European Society of Cardiology (ESC) Guidelines for the management of acute and chronic heart failure [21]. Patients with missing echocardiographic parameters necessary for the calculation of the Virtue Index were excluded from the analysis. The final study cohort consisted of 222 patients, among whom 99 presented with reduced ejection fraction (HFrEF, <40%) and 69 with preserved ejection fraction (HFpEF, ≥50%), whereas individuals with mid-range ejection fraction values (40–49%) were not included. These patients were excluded from both descriptive and prognostic analyses to avoid heterogeneity introduced by an intermediate EF phenotype.

**Figure 1:**
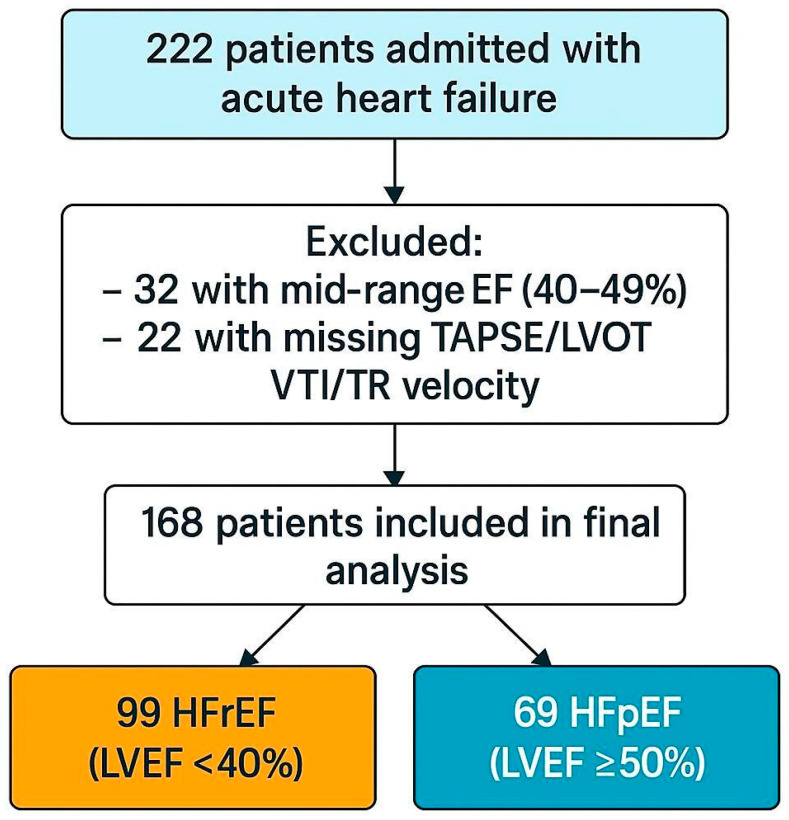
Flow diagram illustrating patient selection and subgroup allocation; Abbreviations: LVEF—left ventricular ejection fraction; HFrEF—heart failure with reduced ejection fraction; HFpEF—heart failure with preserved ejection fraction; TAPSE—tricuspid annular plane systolic excursion; LVOT VTI—left ventricular outflow tract velocity–time integral; TR—tricuspid regurgitation.

The flow of patient selection is illustrated in Figure 1.

### 2.2. Echocardiographic Assessment

Comprehensive transthoracic echocardiography was performed within the first hours after hospital admission, following standard acquisition protocols and current recommendations for chamber quantification and diastolic function assessment. Valvular heart disease was defined as at least moderate dysfunction of one or more valves (aortic stenosis/regurgitation, mitral stenosis/regurgitation, or tricuspid stenosis/regurgitation), according to guideline-recommended echocardiographic criteria [22,23]. All echocardiographic examinations were performed by experienced cardiologists certified in echocardiography, using the same General Electric Vivid E95 (GE Healthcare, Chicago, IL, USA) ultrasound system. The following parameters were assessed: left ventricular ejection fraction (LVEF) by Simpson’s biplane method, tricuspid annular plane systolic excursion (TAPSE) measured in the apical four-chamber view using M-mode [24], left ventricular outflow tract velocity–time integral (LVOT VTI) obtained by pulsed-wave Doppler in the apical five-chamber view [13,14], and the right ventricular-to-right atrial systolic pressure gradient (RV–RA gradient) derived from the peak velocity of tricuspid regurgitation using the modified Bernoulli equation [24]. All measurements were obtained at end-expiration. In patients in sinus rhythm, TAPSE, LVOT VTI and the tricuspid regurgitation–derived RV–RA gradient were calculated as the average of three consecutive cardiac cycles. In patients with atrial fibrillation, we averaged five non–post-ectopic beats with comparable R–R intervals for these parameters to reduce beat-to-beat variability.

The Virtue Index was calculated as: $\frac{RV-RA\;gradient}{TAPSE\times LVOT\;VTI}$.

This index was previously proposed as an integrative marker reflecting the interaction between right ventricular systolic load, longitudinal contractile function, and left ventricular forward flow [20]. In the present study, the same formula was applied to all patients to explore its prognostic significance across different ejection fraction phenotypes.

### 2.3. Biomarker Measurement

Blood samples were obtained at admission, prior to initiation of intravenous therapy. Plasma NT-proBNP concentrations were measured using an electrochemiluminescent immunoassay (Elecsys^®^ proBNP II, Roche Diagnostics, Mannheim, Germany) according to the manufacturer’s instructions. Results were expressed in pg/mL. NT-proBNP was selected as a comparator biomarker due to its well-established diagnostic and prognostic significance in AHF, as demonstrated in several pivotal studies [25,26,27].

### 2.4. Outcomes

The primary objective of the study was to evaluate the prognostic performance of the Virtue Index for in-hospital all-cause mortality.

Secondary analyses included assessment of the relationship between the Virtue Index and NT-proBNP at admission and comparison of its discriminative ability with conventional echocardiographic parameters (TAPSE, RV–RA gradient, and LVOT VTI).

### 2.5. Statistical Analysis

Continuous variables were expressed as mean ± standard deviation or median (interquartile range), as appropriate. Comparisons between groups used the Student *t*-test for normally distributed data [28] or the Mann–Whitney U test for non-normally distributed variables [29]. Categorical variables were compared with the χ^2^ test.

Associations between the Virtue Index and NT-proBNP were evaluated using Spearman’s rank correlation coefficient, with 1000 bootstrap replicates to obtain 95% confidence intervals. Prognostic performance for in-hospital mortality was assessed with receiver operating characteristic (ROC) analysis and area under the curve (AUC), with bootstrap confidence intervals. Pairwise comparisons of AUC values were performed using DeLong’s test for correlated ROC curves [30].

Left ventricular ejection fraction (LVEF) was not included in the ROC analysis because it served as the primary stratification variable defining the two EF phenotypes (HFrEF and HFpEF). Including LVEF as an independent predictor within EF-based subgroups would introduce circularity and artificially inflate its apparent prognostic performance.

Given the low number of in-hospital deaths, multivariable logistic regression was not performed to avoid substantial overfitting. Instead, confounding was mitigated through EF-phenotype stratification (HFrEF vs. HFpEF) and by reporting univariate effect sizes with 95% bootstrap confidence intervals.

All statistical tests were two-tailed, and a *p*-value < 0.05 was considered significant. Analyses were performed using R (version 4.3.2; R Foundation for Statistical Computing, Vienna, Austria) and Python (version 3.10; Python Software Foundation, Wilmington, DE, USA).

Generative artificial intelligence tools (ChatGPT, version GPT-5.1, OpenAI, San Francisco, CA, USA) were used for figure generation and minor linguistic adjustments to improve text clarity and readability.

## 3. Results

### 3.1. Baseline Characteristics

Baseline demographic, clinical, and laboratory characteristics were compared between patients with reduced (HFrEF) and preserved ejection fraction (HFpEF). Detailed results are presented in Table 1.

Regarding EF-based acute heart failure phenotypes, 58.9% of patients had HFrEF and 41.1% had HFpEF (Table 1). These proportions served as the basis for subgroup prognostic comparisons.

Patients with HFpEF were significantly older than those with HFrEF (77.6 ± 9.6 vs. 65.9 ± 14.9 years, *p* < 0.001) and were more frequently female (72.5% vs. 33.3%, *p* < 0.001). Smoking was considerably less common in the HFpEF group (8.7% vs. 36.4%, *p* < 0.001). Hypertension was more prevalent in HFpEF (95.7% vs. 82.8%, *p* = 0.017), while dyslipidemia showed a similar prevalence across groups (94.2% vs. 94.9%, *p* = 0.83). Obesity (24.6% vs. 30.3%, *p* = 0.46) and diabetes mellitus (37.7% vs. 41.4%, *p* = 0.63) did not differ significantly between groups. Valvular heart disease was slightly more common in HFrEF (80.8% vs. 73.9%, *p* = 0.29).

Hemodynamic parameters were broadly comparable, with no significant difference in systolic blood pressure (143 ± 31 in HFrEF vs. 142 ± 31 mmHg in HFpEF, *p* = 0.83), while diastolic pressure was slightly higher in HFrEF (87 ± 17 vs. 81 ± 17 mmHg, *p* = 0.02). Median NT-proBNP levels were numerically higher in HFrEF (8453 [4957–21,121] pg/mL) than in HFpEF (6537 [2180–24,554] pg/mL), though the difference was not statistically significant (*p* = 0.41).

Echocardiographic findings were consistent with the expected phenotypic pattern. TAPSE values were similar between groups (20 ± 10 mm vs. 21 ± 7 mm, *p* = 0.41), whereas LVOT VTI was markedly lower in HFrEF (14 ± 4 cm vs. 21 ± 7 cm, *p* < 0.001). As anticipated, LVEF differed profoundly (28 ± 7% vs. 60 ± 7%, *p* < 0.001). The RV–RA gradient was significantly higher in HFpEF (21 [17–24] mmHg) compared with HFrEF (17 [14–22] mmHg, *p* < 0.001), reflecting greater pulmonary pressure load.

The Virtue Index, reflecting the integrated right–left ventricular interaction, was significantly higher in HFrEF than in HFpEF [0.135 (0.069–0.215) vs. 0.075 (0.049–0.110), *p* < 0.001], consistent with the more pronounced systolic impairment characterizing the reduced-EF phenotype.

### 3.2. Prognostic Discrimination for In-Hospital Mortality (ROC/AUC Analyses)

We next evaluated the discriminative ability of the Virtue Index and conventional echocardiographic parameters: RV–RA gradient, TAPSE, and LVOT VTI—for in-hospital mortality.

In the HFrEF subgroup (n = 99, 9 (9%) deaths), Virtue showed modest discrimination (AUC 0.584, 95% CI 0.36–0.79), similar to RV–RA gradient (0.532, 95% CI 0.46–0.72) and TAPSE (0.583, 95% CI 0.45–0.77). LVOT VTI performed best in this group (AUC 0.700, 95% CI 0.53–0.85).

In the HFpEF subgroup (n = 69, 8 (11.6%) deaths), Virtue achieved good discrimination (AUC 0.704, 95% CI 0.53–0.85), comparable to RV–RA gradient (0.724, 95% CI 0.54–0.90) and higher than TAPSE (0.637, 95% CI 0.45–0.89) and LVOT VTI (0.669, 95% CI 0.45–0.86).

**Figure 2:**
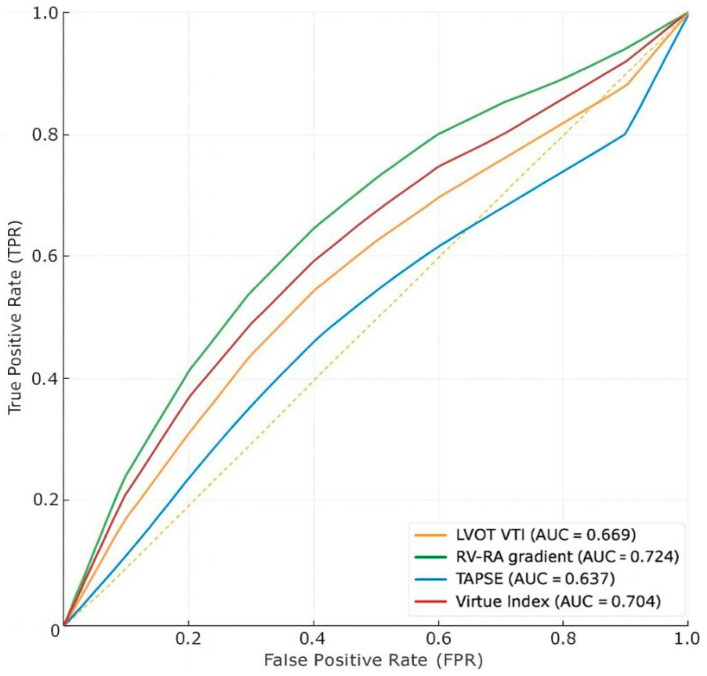
Receiver operating characteristic (ROC) curves illustrating the predictive performance of the Virtue Index and conventional echocardiographic parameters for in-hospital mortality in patients with HFpEF. RV–RA gradient = right ventricular to right atrial pressure gradient; TAPSE = tricuspid annular plane systolic excursion; LVOT VTI = left ventricular outflow tract velocity–time integral; Yellow dotted line = reference line (AUC = 0.5).

**Figure 3:**
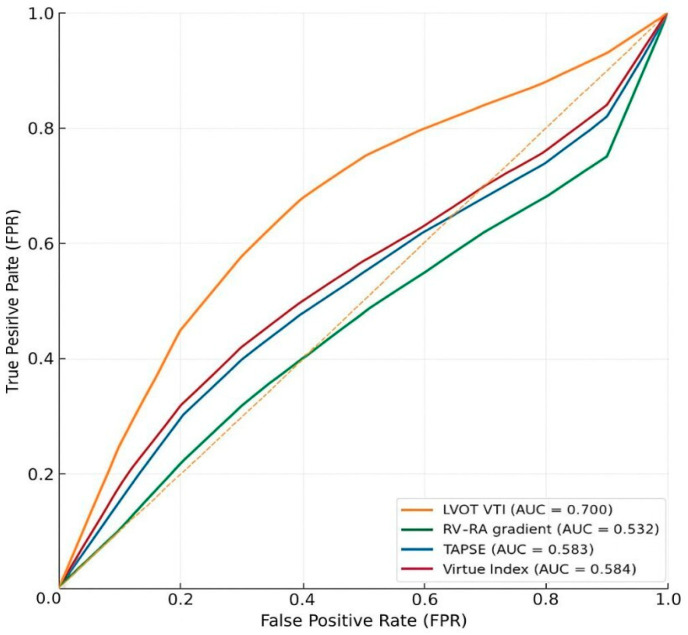
Receiver operating characteristic (ROC) curves illustrating the predictive performance of the Virtue Index and conventional echocardiographic parameters for in-hospital mortality in patients with HFrEF. RV–RA gradient = right ventricular to right atrial pressure gradient; TAPSE = tricuspid annular plane systolic excursion; LVOT VTI = left ventricular outflow tract velocity–time integral; Yellow dotted line = reference line (AUC = 0.5).

Numerical results are shown in Table 2, while Figure 2 and Figure 3 provide graphical representations of the AUC values and ROC curves for each subgroup.

#### Interpretation

In the HFrEF subgroup, the Virtue Index demonstrated only modest discrimination for in-hospital mortality (AUC 0.584), similar to TAPSE and RV–RA gradient—parameters traditionally recognized as limited short-term prognostic markers in advanced systolic heart failure. LVOT VTI showed the highest predictive accuracy (AUC 0.700), underscoring the dominant role of left ventricular stroke volume and forward flow in determining outcomes when systolic function is severely reduced. Thus, in patients with reduced EF, Virtue does not appear to provide additional prognostic information beyond established systolic indices.

Conversely, in the HFpEF subgroup, Virtue achieved good discrimination (AUC 0.704), comparable to RV–RA gradient (AUC 0.724) and clearly outperforming TAPSE and LVOT VTI. Its significant correlation with NT-proBNP supports its physiological relevance as an integrative marker of right–left ventricular interaction and filling pressures. In this context, where conventional systolic indices often fail to predict outcomes, composite measures such as Virtue may more accurately capture the hemodynamic determinants of prognosis.

Overall, the prognostic performance of Virtue appears phenotype-dependent—limited in HFrEF, where LV forward flow remains the main determinant of short-term outcomes, but more informative in HFpEF, reflecting the interplay between right-sided pressures, longitudinal function, and LV outflow.

### 3.3. Correlation Between Virtue Index and NT-proBNP

In subgroup analyses, the Virtue Index demonstrated distinct patterns of correlation with NT-proBNP levels at admission.

In the HFrEF subgroup, the correlation was weak and not statistically significant (ρ = 0.191, 95% CI −0.006–0.39, *p* = 0.06; n = 96 pairs). This suggests that in patients with reduced ejection fraction, where neurohormonal activation and structural remodeling are typically advanced, Virtue may add limited prognostic information beyond NT-proBNP.

In contrast, in the HFpEF subgroup, Virtue showed a moderate and statistically significant correlation (ρ = 0.380, 95% CI 0.13–0.58, *p* = 0.002; n = 66 pairs). This supports the biological plausibility of Virtue as an integrated marker of congestion and ventricular interaction in patients with preserved systolic function, where conventional indices often fail to capture relevant prognostic information.

These results are summarized in Table 3. Taken together, they indicate that the relationship between Virtue and NT-proBNP may be phenotype-dependent: weak in reduced EF, but stronger and clinically relevant in preserved EF, supporting its potential role as an integrated hemodynamic marker in HFpEF.

### 3.4. Pairwise AUC Comparisons Between Virtue and Conventional Parameters

The discriminative ability of Virtue was directly compared with RV–RA gradient, TAPSE, and LVOT VTI using pairwise AUC differences (Hanley–McNeil approximation of the DeLong test). The results are summarized in Table 4.

In the HFrEF subgroup (n = 99, 9 deaths −9.1%), Virtue demonstrated marginally higher AUCs than the RV–RA gradient (ΔAUC = +0.052, Z = 0.407, *p* = 0.684) and TAPSE (ΔAUC = +0.001, Z = 0.008, *p* = 0.994) but performed slightly worse than LVOT VTI (ΔAUC = −0.116, Z = −0.894, *p* = 0.372). None of these differences reached statistical significance.

In the HFpEF subgroup (n = 69, 8 deaths −11.6%), Virtue showed comparable discrimination to the RV–RA gradient (ΔAUC = −0.020, Z = −0.186, *p* = 0.852) and modestly higher AUCs than TAPSE (ΔAUC = +0.067, Z = 0.504, *p* = 0.614) and LVOT VTI (ΔAUC = +0.035, Z = 0.277, *p* = 0.782). Again, none of these pairwise comparisons were statistically significant.

#### Interpretation

In HFrEF, the Virtue Index performed similarly to RV–RA gradient and TAPSE, while LVOT VTI remained the most discriminative parameter, consistent with its established role as a marker of forward stroke volume and systolic output.

In HFpEF, Virtue exhibited comparable or slightly superior discrimination relative to conventional indices. Although these differences were not statistically significant, the index appears to integrate aspects of ventricular coupling and congestion more effectively in this phenotype.

Overall, the prognostic performance of the Virtue Index was modest. In HFpEF, Virtue showed a trend toward better alignment with congestion burden and performed similarly to established echocardiographic indices. In HFrEF, where global systolic dysfunction predominates, its contribution was limited and LVOT VTI remained the most accurate predictor.

### 3.5. Comparative Prognostic Performance of Virtue and NT-proBNP

We further compared the discriminative performance of the Virtue Index with NT-proBNP at admission.

In HFrEF (n = 99, 9 deaths −9.1%), Virtue achieved AUC 0.583, while NT-proBNP reached 0.744, confirming the superior prognostic accuracy of the biomarker in systolic dysfunction.

In HFpEF (n = 69, 8 deaths −11.6%) Virtue reached AUC 0.705, whereas NT-proBNP achieved 0.838, again outperforming the echocardiographic index in short-term mortality prediction.

**Figure 4:**
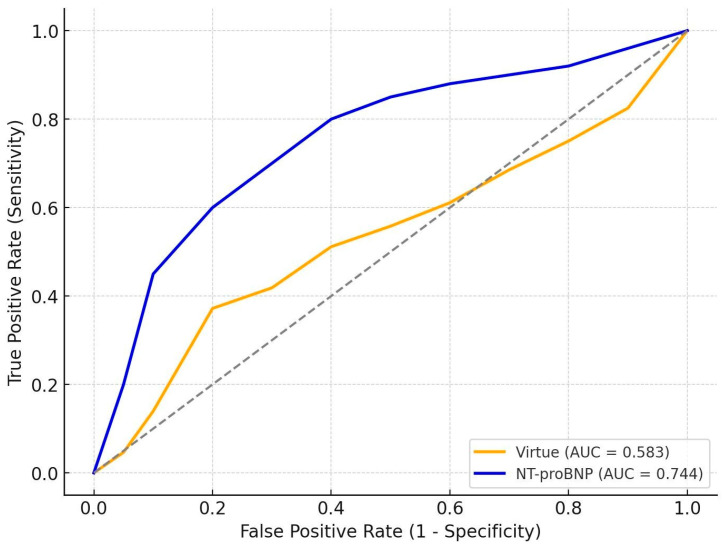
Receiver operating characteristic (ROC) curves comparing the Virtue Index and NT-proBNP for in-hospital mortality prediction in patients with HFrEF; Dotted line = reference line (AUC = 0.5).

**Figure 5:**
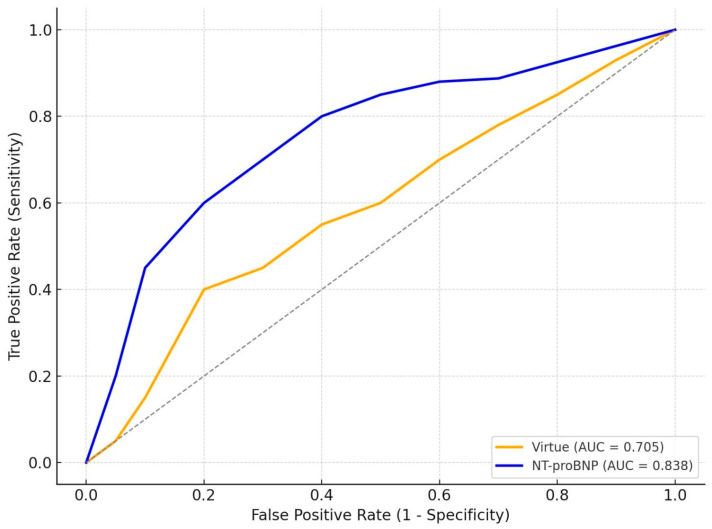
Receiver operating characteristic (ROC) curves comparing the Virtue Index and NT-proBNP for in-hospital mortality prediction in patients with HFpEF; Dotted line = reference line (AUC = 0.5).

Numerical results are summarized in Table 5, and graphical representations of the ROC curves are shown in Figure 4 and Figure 5.

#### Interpretation

Across both subgroups, NT-proBNP generally provided superior prognostic discrimination compared with the Virtue Index. In HFrEF, NT-proBNP showed substantially better performance (AUC 0.744 vs. 0.583, *p* = 0.04), underscoring its established role as a powerful marker of neurohormonal activation and decompensation in systolic heart failure. In HFpEF, Virtue achieved reasonable discrimination (AUC 0.705), although NT-proBNP remained superior (AUC 0.838, *p* = 0.05).

These findings suggest that while Virtue captures relevant hemodynamic information, NT-proBNP retains higher accuracy as a standalone prognostic marker in both phenotypes. Importantly, the comparable performance of Virtue in HFpEF supports its potential complementary role, particularly when echocardiography provides immediate bedside insights and NT-proBNP results may be delayed or unavailable.

## 4. Discussion

In the present study, we aimed to validate the Virtue Index in a cohort of patients admitted with acute heart failure (AHF) and to assess its prognostic significance across phenotypes defined by left ventricular ejection fraction (LVEF). Our findings indicate that the Virtue Index retains prognostic value, although its performance varies with phenotype. Specifically, it provided stronger discrimination and closer alignment with NT-proBNP in HFpEF, whereas its contribution was more modest in HFrEF, where parameters of forward flow dominated outcome prediction. These results extend previous observations and emphasize the importance of integrating ventricular mechanics and congestion profiles when interpreting echocardiographic prognostic markers [31,32,33].

The heterogeneity of AHF continues to complicate risk assessment and management. While systolic dysfunction and reduced cardiac output characterize HFrEF, diastolic stiffness, abnormal relaxation, and ventriculo-arterial uncoupling define HFpEF [31,34]. These distinct mechanisms influence not only congestion patterns and filling pressures but also the prognostic interpretation of imaging and biomarker parameters. The Virtue Index—combining tricuspid regurgitation gradient with TAPSE and LVOT VTI—was conceived to capture this complex interaction between right- and left-sided function. By linking pulmonary pressure load to longitudinal contractility and stroke volume, it integrates the efficiency of biventricular coupling and the hemodynamic cost of maintaining cardiac output [13,18,35]. This aligns with the physiological concept that in AHF, forward flow and venous congestion are tightly interdependent: TAPSE primarily reflects RV contractile reserve, the RV–RA gradient represents RV afterload from pulmonary pressures, while LVOT VTI estimates effective systemic perfusion. Their integration into a single index therefore provides a mechanistic marker of acute circulatory compromise that may not be captured by LVEF alone.

The clinical profile of our cohort illustrates the contrasting characteristics of heart failure phenotypes [31,34]. Patients with HFpEF were typically older, more often women, and had a greater prevalence of atrial fibrillation, hypertension, and renal dysfunction compared with those with reduced ejection fraction [31,32,33]. Despite preserved systolic function, these patients experienced higher in-hospital mortality. The combination of advanced age, multiple comorbidities, and impaired diastolic reserve likely creates a fragile physiological balance that is easily disrupted during acute decompensation [32,34]. Increased vascular stiffness, endothelial dysfunction, and altered ventricular–vascular coupling further limit cardiac adaptability to volume and pressure overload, resulting in greater vulnerability to congestion and instability [32,36].

The frequent coexistence of atrial fibrillation in HFpEF adds to this complexity by reducing atrial contribution to ventricular filling and increasing pulmonary pressures [33,36]. When combined with hypertension, renal impairment, and age-related vascular changes, these factors delineate a high-risk profile that explains the observed trend toward greater in-hospital mortality in HFpEF despite preserved systolic performance [31,32,33,34,35,36].

Within this context, the interaction between right and left ventricular function appears particularly relevant in HFpEF. In this subgroup, the Virtue Index correlated significantly with NT-proBNP and achieved comparable prognostic performance to the RV–RA gradient, outperforming TAPSE and LVOT VTI. These findings are consistent with reports highlighting the central role of ventricular interdependence and pulmonary pressure adaptation in preserved-EF syndromes [32,36]. In HFpEF, even small increases in left ventricular filling pressure can lead to disproportionate rises in pulmonary artery pressure and early right ventricular dysfunction [33,36]. Consequently, both Virtue and NT-proBNP may reflect overlapping pathophysiological domains—specifically diastolic load, wall stress, and venous congestion—rather than isolated contractile impairment. This alignment supports Virtue as a feasible echocardiographic surrogate for congestion in settings where biomarker testing is delayed or unavailable [20,37].

In contrast, Virtue showed limited predictive ability in HFrEF, where LVOT VTI emerged as the most powerful echocardiographic predictor. This observation aligns with the established pathophysiology of systolic heart failure, in which stroke volume and forward output remain the principal determinants of short-term outcomes [11,12,22]. As systolic dysfunction advances, the variability of TAPSE and tricuspid gradients narrows, diminishing the discriminative capacity of composite coupling indices. Similar attenuation has been reported for other metrics such as cardiac power output and RV/LV interaction ratios, whose incremental prognostic value declines once left ventricular contractility becomes severely impaired [35,38].

In our cohort, LVEF was not significantly associated with in-hospital mortality (Table 1), in line with prior evidence showing its limited short-term prognostic value in acute heart failure. Therefore, assessing discrimination within EF-based subgroups appropriately focuses on parameters other than LVEF to avoid circular interpretation.

As expected, NT-proBNP outperformed all echocardiographic indices in both phenotypes. In absolute terms, higher Virtue Index values reflect a hemodynamically unfavorable condition, where right-sided systolic pressure load disproportionately exceeds effective forward stroke volume, indicating impaired ventriculo–pulmonary coupling. Conversely, lower values suggest a more efficient interaction between the ventricles and pulmonary circulation. The alignment of high Virtue values with elevated NT-proBNP levels further supports this interpretation, as NT-proBNP ≥ 300 pg/mL is widely accepted as a diagnostic threshold for acute heart failure and reflects significant hemodynamic stress and congestion [8,9,10]. However, in our cohort, NT-proBNP values were markedly elevated in both phenotypes (median > 6000 pg/mL), consistent with the severity of hemodynamic decompensation at admission; therefore, the 300 pg/mL threshold serves primarily as a clinical reference rather than a discriminatory cut-off in this setting. Although NT-proBNP retained superior discriminatory power, Virtue provides an immediate imaging-based estimate of hemodynamic burden, particularly in acute presentations where biomarker confirmation may be delayed, and may therefore support rapid bedside prognostic assessment [34,39].

Methodologically, this study expands on the initial validation of Virtue [20] by analyzing a larger, more heterogeneous population and incorporating phenotype-specific assessment with bootstrap-derived confidence intervals. The absence of significant differences in DeLong testing likely reflects the limited number of events; nevertheless, the consistent direction of results across parameters supports the comparable performance of Virtue to established echocardiographic predictors derived from routine measurements.

Several limitations should be acknowledged. The retrospective, single-center design may introduce selection bias. Given the very low number of events, multivariable modelling was not feasible without causing major overfitting and unstable estimates. The limited number of events also reduces the precision of prognostic estimates, which is reflected by the wide confidence intervals and the lack of statistically significant differences in some pairwise ROC comparisons. Therefore, the findings should be interpreted as exploratory and hypothesis-generating rather than definitive.

Another important limitation is that echocardiography was performed early after admission, before complete hemodynamic stabilization. Loading conditions may therefore have influenced TAPSE, LVOT VTI and the TR-derived RV–RA gradient. As a retrospective study, detailed information on timing relative to diuretic therapy, congestion markers, or dynamic evolution of volume status was not consistently available. Consequently, we could not adjust the prognostic analyses for loading conditions, and this may have influenced the observed effect sizes.

Moderate and severe valvular dysfunction may have influenced the accuracy of TR-derived gradients and LVOT VTI measurements, given their known dependence on loading conditions.

NT-proBNP values were measured only at admission, precluding assessment of dynamic changes. Prospective multicenter validation with longitudinal follow-up is warranted to confirm these findings and to determine whether serial Virtue measurements can track therapeutic response or predict post-discharge outcomes.

Despite these limitations, the present analysis provides new insight into phenotype-specific prognostication in AHF. Virtue appears to capture the integrated hemodynamic burden that drives outcomes in HFpEF, whereas in HFrEF its prognostic value is overshadowed by global systolic failure. This phenotype-dependent pattern supports a combined approach to risk stratification—one that integrates biomarkers and echocardiographic indices rather than considering them competing tools. Future research should explore whether combining Virtue with NT-proBNP or other congestion markers could enhance real-time risk assessment and optimize bedside decision-making in acute heart failure [34,36,37,38,39].

Although the index showed prognostic relevance, especially in HFpEF, its discriminatory performance was only moderate and several comparisons with established parameters did not reach statistical significance.

## 5. Conclusions

The prognostic performance of the VIRTUE Index was modest overall and phenotype-dependent. In HFpEF, the RV–RA gradient performed slightly better than the index, although it showed a closer association with NT-proBNP levels and a better, albeit still limited, ability to identify patients at higher short-term risk, likely reflecting the combined effects of congestion and ventricular interaction [20,31,33,36]. In HFrEF, simpler measures of forward flow such as LVOT VTI remained clearly superior for predicting in-hospital mortality [11,12,22].

Overall, these findings remain exploratory and hypothesis-generating, and the VIRTUE Index should currently be regarded as a potential complement to established prognostic tools rather than a replacement. Larger, prospective studies are required to validate its clinical relevance and assess whether serial changes could help refine risk stratification in AHF [20,35,39].

## Figures and Tables

**Table 1:** Baseline demographic, clinical, and echocardiographic characteristics according to ejection fraction phenotype.

Variable	Total (n = 168)	HFrEF (n = 99)	HFpEF (n = 69)	*p*-Value
Age (years)	71 ± 14	65.9 ± 14.9	77.6 ± 9.6	<0.001
Male sex, %	50.6%	66.7%	27.5%	<0.001
Smoking, %	25.5%	36.4%	8.7%	<0.001
Hypertension, %	88.1%	82.8%	95.7%	0.017
Dyslipidemia, %	94.6%	94.9%	94.2%	0.830
Obesity, %	28.1%	30.3%	24.6%	0.460
Diabetes mellitus, %	39.9%	41.4%	37.7%	0.630
Valvular heart disease, %	78.0%	80.8%	73.9%	0.290
Systolic BP (mmHg)	143 ± 31	143 ± 31	142 ± 31	0.837
Diastolic BP (mmHg)	85 ± 17	87 ± 17	81 ± 17	0.026
NT-proBNP (pg/mL)	7830 (3698–21,764)	8453 (4957–21,121)	6537 (2180–24,554)	0.411
RV–RA gradient (mmHg)	30.0 (22.8–40.0)	17.0 (14.0–22.0)	21.0 (17.0–24.0)	<0.001
TAPSE (mm)	21 ± 9	20 ± 10	21 ± 7	0.410
LVOT VTI (cm)	16 ± 5	14 ± 4	21 ± 7	<0.001
LVEF (%)	41 ± 18	28 ± 7	60 ± 7	<0.001
Atrial fibrillation	47.0%	36.4%	62.3%	0.002
CAD	45.8%	58.6%	27.5%	<0.001
eGFR at admission	60.0 (44.8–81.0)	63.0 (46.0–81.5)	54.0 (44.0–80.0)	0.030
In-hospital mortality	10.1%	9.1%	11.6%	0.788
Virtue Index	0.098 (0.057–0.190)	0.135 (0.069–0.215)	0.075 (0.049–0.110)	<0.001

Data are presented as mean ± standard deviation (SD) for normally distributed variables or as median [interquartile range] for non-normally distributed variables. Categorical variables are expressed as percentages. Comparisons were performed between patients with reduced ejection fraction (HFrEF, LVEF < 40%) and those with preserved ejection fraction (HFpEF, LVEF ≥ 50%). *p*-values refer to differences between HFrEF and HFpEF. BP = blood pressure; CAD = coronary artery disease; eGFR = estimated glomerular filtration rate; HFrEF—heart failure with reduced ejection fraction; HFpEF—heart failure with preserved ejection fraction; LVOT VTI = left ventricular outflow tract velocity–time integral; LVEF = left ventricular ejection fraction; NT-proBNP = N-terminal pro–B-type natriuretic peptide; RV–RA = right ventricular to right atrial pressure gradient; TAPSE = tricuspid annular plane systolic excursion; Valvular heart disease: ≥moderate dysfunction of any cardiac valve.

**Table 2:** Receiver operating characteristic (ROC) analysis of echocardiographic parameters for in-hospital mortality.

Group	Predictor	n	Events	AUC	CI 95%	*p*-Value
HFrEF	Virtue Index	99	9	0.584	0.364 to 0.791	0.441
HFrEF	RV–RA gradient	105	9	0.532	0.463 to 0.724	0.631
HFrEF	TAPSE	105	9	0.583	0.454 to 0.770	0.303
HFrEF	LVOT VTI	102	9	0.700	0.530 to 0.850	0.014
HFpEF	Virtue Index	69	8	0.704	0.536 to 0.852	0.011
HFpEF	RV–RA gradient	76	8	0.724	0.542 to 0.902	0.015
HFpEF	TAPSE	76	8	0.637	0.457 to 0.891	0.216
HFpEF	LVOT VTI	71	8	0.669	0.456 to 0.861	0.102

Area under the curve (AUC) values with 95% confidence intervals (CI) for the Virtue Index and conventional echocardiographic parameters, stratified by ejection fraction (EF); Differences in N reflect missing data for specific echocardiographic variables; AUC = area under the ROC curve; CI = confidence interval. HFrEF—heart failure with reduced ejection fraction; HFpEF—heart failure with preserved ejection fraction; RV–RA = right ventricular to right atrial pressure gradient; TAPSE = tricuspid annular plane systolic excursion; LVOT VTI = left ventricular outflow tract velocity–time integral.

**Table 3:** Spearman correlation coefficients (ρ) between the Virtue Index and NT-proBNP, stratified by ejection fraction (EF) subgroup, with 95% confidence intervals (CI).

Group	N_Pairs	Spearman_rho	CI 95%	*p*-Value
HFrEF	96	0.191	−0.006 to 0.384	0.061
HFpEF	66	0.380	0.134 to 0.580	0.002

Values are presented as Spearman correlation coefficients (ρ) with 95% confidence intervals (CI); HFrEF—heart failure with reduced EF; HFpEF—heart failure with preserved EF.

**Table 4:** Pairwise AUC comparisons between the Virtue Index and conventional echocardiographic parameters using the DeLong test, stratified by ejection fraction (EF) subgroup.

Group	Comparison	Delta_AUC (Virtue—X)	Z	*p*-Value	N_Common
HFrEF	Virtue vs. RV–RA gradient	0.052	0.407	0.684	99
HFrEF	Virtue vs. TAPSE	0.001	0.008	0.994	99
HFrEF	Virtue vs. LVOT VTI	−0.116	−0.894	0.372	99
HFpEF	Virtue vs. RV–RA gradient	−0.020	−0.186	0.852	69
HFpEF	Virtue vs. TAPSE	0.067	0.504	0.614	69
HFpEF	Virtue vs. LVOT VTI	0.035	0.277	0.782	69

AUC = area under the ROC curve; ΔAUC = difference in AUC between Virtue and comparator; HFrEF = heart failure with reduced ejection fraction; HFpEF = heart failure with preserved ejection fraction. RV–RA = right ventricular to right atrial pressure gradient; TAPSE = tricuspid annular plane systolic excursion; LVOT VTI = left ventricular outflow tract velocity–time integral; N_common = number of paired observations included in both ROC analyses; Z = standardized Z-score derived from the DeLong test.

**Table 5:** Comparison of area under the ROC curve (AUC) values for the Virtue Index and NT-proBNP in predicting in-hospital mortality, stratified by ejection fraction (EF) subgroup.

Group	n	AUC_Virtue	AUC_NT-proBNP	*p*-Value
HFrEF	99	0.583	0.744	0.040
HFpEF	69	0.705	0.838	0.050

AUC = area under the ROC curve; HFrEF—heart failure with reduced ejection fraction; HFpEF—heart failure with preserved ejection fraction; NT-proBNP = N-terminal pro–B-type natriuretic peptide; n = number of patients included in each subgroup.

## Data Availability

The data supporting the findings of this study are available from the corresponding author upon reasonable request. Due to patient privacy regulations, the dataset cannot be made publicly accessible.

## Abbreviations

The following abbreviations are used in this manuscript:

AUC	Area Under the Curve
AHF	Acute Heart Failure
BP	Blood Pressure
CAD	Coronary Artery Disease
CI	Confidence Interval
EF	Ejection Fraction
ESC	European Society of Cardiology
eGFR	Estimated Glomerular Filtration Rate
HFpEF	Heart Failure with Preserved Ejection Fraction
HFrEF	Heart Failure with Reduced Ejection Fraction
IQR	Interquartile Range
LV	Left Ventricle
LVEF	Left Ventricular Ejection Fraction
LVOT VTI	Left Ventricular Outflow Tract Velocity Time Integral
NT-proBNP	N-terminal pro B-type Natriuretic Peptide
ROC	Receiver Operating Characteristic
RV	Right Ventricle
RV–RA gradient	Right Ventricular to Right Atrial Pressure Gradient
SD	Standard Deviation
TAPSE	Tricuspid Annular Plane Systolic Excursion
